# Auricular Vagus Nerve Stimulation in Inflammatory Bowel Disease: Mechanistic Insights and Biomarker-Based Evaluation of Therapeutic Response

**DOI:** 10.3390/ijms27156958

**Published:** 2026-08-03

**Authors:** Elif Zeynep Öztürk, Mehlika Alataş

**Affiliations:** 1 Department of Fundamental Sciences, Faculty of Engineering and Natural Sciences, Kutahya Health Science University, Kütahya 43100, Turkey; 2 Department of Physical Therapy and Rehabilitation, Faculty of Health Sciences, Bahçeşehir University, İstanbul 34353, Turkey

**Keywords:** Crohn’s disease, gut–brain–immune axis, inflammatory bowel disease, ulcerative colitis, transcutaneous auricular vagus nerve stimulation

## Abstract

Inflammatory bowel disease (IBD), encompassing Crohn’s disease (CD) and ulcerative colitis (UC), is a chronic inflammatory disorder characterized by dysregulated host–microbiota interactions, epithelial barrier dysfunction, and aberrant immune activation. Despite advances in biologic and small-molecule therapies, a substantial proportion of patients exhibit incomplete response, loss of response, or treatment-related adverse effects, underscoring the need for novel adjunctive therapeutic approaches. The vagus nerve exerts a pivotal effects on immune homeostasis through the cholinergic anti-inflammatory pathway, thereby integrating peripheral immune sensing with central autonomic regulation. Within the context of IBD, a diminution in vagal tone has been observed to correlate with persistent mucosal inflammation and immune dysregulation. Transcutaneous auricular vagus nerve stimulation (ta-VNS) is a non-invasive neuromodulation technique that may activate vagal circuits and modulate neuroimmune signaling without surgical intervention. This review endeavors to critically evaluate the potential efficacy of ta-VNS in the context of IBD, with particular emphasis on its discernible effects on immune, epithelial barrier, microbiota-related, and autonomic biomarkers. Emerging evidence, stemming from preliminary investigations, suggests that ta-VNS may exert an influence on inflammatory cytokine profiles, epithelial tight junction integrity, gut microbial composition, and systemic autonomic balance, warranting further elucidation. In addition, the review discusses differential responses between Crohn’s disease and ulcerative colitis, summarizes current preclinical and clinical evidence, and highlights biomarker-based strategies for evaluating therapeutic response in future randomized controlled trials. Although current evidence remains preliminary and stimulation parameters are not yet standardized, ta-VNS represents a promising adjunctive neuroimmune modulation strategy in IBD.

## 1. Introduction

Inflammatory bowel diseases (IBD), primarily comprising Crohn’s disease (CD) and ulcerative colitis (UC), are chronic immune-mediated disorders of the gastrointestinal tract [1,2]. These diseases affect millions of individuals worldwide and commonly manifest during adolescence or early adulthood [2]. Specifically, CD is characterized by transmural, Th1/Th17-mediated inflammation capable of affecting any segment of the gastrointestinal tract (commonly the terminal ileum and colon); conversely, UC presents as superficial mucosal inflammation restricted to the colon and rectum [3]. The etiology of IBD is multifaceted, involving genetic susceptibility, dysregulation of innate and adaptive immune responses, environmental determinants (e.g., smoking, dietary patterns), and gut dysbiosis, all of which contribute to its pathogenesis [4,5,6]. Despite advances in biologic and small-molecule therapies, many patients experience inadequate response, secondary loss of response, or treatment-related adverse effects, highlighting the need for novel adjunctive therapeutic strategies [7]. Specifically, anti-TNF therapy, often considered a cornerstone treatment, may induce remission in a limited subset of patients, with a reported 23–46% experiencing loss of response over time [8]. Treatment costs, adverse effects, secondary loss of response, and difficulties with long-term adherence further contribute to the clinical and socioeconomic burden of IBD [9,10,11]. These limitations underscore the pressing need for safe, non-pharmacological therapeutic strategies as adjunctive treatment options.

The vagus nerve plays a central role in immune homeostasis through activation of the cholinergic anti-inflammatory pathway, integrating peripheral immune sensing with central autonomic regulation [12]. Vagal efferent fibers effect the release of acetylcholine (ACh), the binding of which to α7 nicotinic acetylcholine receptors, expressed on macrophages and other immune cells, demonstrably suppresses the production of pro-inflammatory cytokines, including tumor necrosis factor-α (TNF-α), interleukin-1β, and interleukin-6. This suppressive action constitutes a component of a broader mechanism, formally termed the cholinergic anti-inflammatory pathway (CAIP) [8,12].

Concurrently, vagal afferents detect peripheral inflammatory signals, including circulating cytokines, and transmit this information to central autonomic nuclei, such as the nucleus tractus solitarius (NTS), which subsequently activate higher brain centers; these centers, in turn, engage both the hypothalamic–pituitary–adrenal (HPA) axis—thereby eliciting an increase in cortisol secretion—and descending vagal efferent pathways [13]. Collectively, these bidirectional circuits constitute the so-called inflammatory reflex, a neural feedback system essential for the constraint of excessive immune activation in the gut [12,13,14]. Under physiological conditions, vagal activity contributes to epithelial barrier integrity, limits microbial translocation, and promotes immunoregulatory pathways [15].

In the context of IBD, mounting evidence suggests an underlying autonomic imbalance. Individuals diagnosed with UC frequently manifest a reduction in parasympathetic (vagal) tone, whilst those diagnosed with CD often exhibit a relative sympathetic predominance [16,17]. Lindgren and colleagues reported that individuals with CD manifested impaired sympathetic regulation, whereas those with UC demonstrated diminished vagal activity when compared with healthy controls [18]. Furthermore, elevated circulating TNF levels in CD have been demonstrated to evince an inverse correlation with vagal tone. Furthermore, experimental vagotomy appears to increase susceptibility to intestinal inflammation in animal models [1].

Cigarette smoking—and specifically nicotine, a cholinergic agonist—exerts a paradoxical protective effect in UC but not in CD, further implicating vagal–ACh signaling in UC pathophysiology [1]. Taken together, these findings suggest that impaired autonomic regulation may contribute to intestinal inflammation in both CD and UC; however, the available evidence is insufficient to establish consistent disease-specific differences.

**Figure 1:**
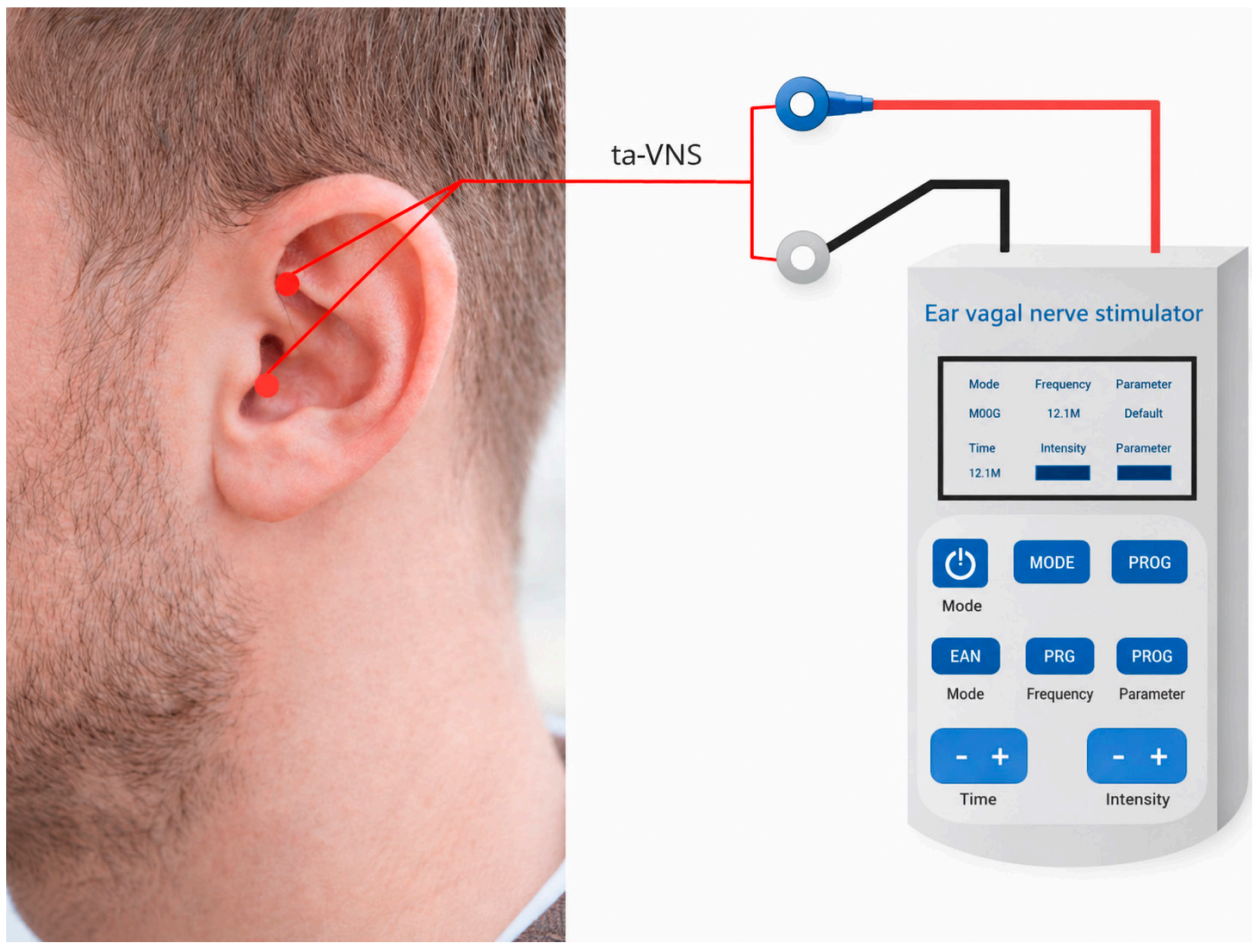
Transcutaneous auricular VNS (ta-VNS). This figure was generated using an artificial intelligence-assisted image-generation tool and was subsequently reviewed and revised by the authors for scientific accuracy.

These findings provide a rationale for investigating vagus nerve stimulation as a potential adjunctive therapy for IBD. Invasive cervical VNS—a modality involving an implanted electrode—has received FDA approval for the treatment of epilepsy and depression. Preliminary investigations suggest that implanted VNS reduces disease activity and biomarkers (e.g., fecal calprotectin, TNF) in patients with IBD [19]. Nonetheless, the application of invasive cervical VNS is not devoid of inherent risks. ta-VNS non-invasively stimulates the auricular branch of the vagus nerve, thereby avoiding the need for surgical implantation (Figure 1) [8]. Commercial ta-VNS devices are available and are generally considered well tolerated. Demonstrated anti-inflammatory effects of ta-VNS have been observed in other conditions, such as rheumatoid arthritis, dyspepsia, and IBS [20,21,22,23]; consequently, it is posited that ta-VNS may similarly activate these anti-inflammatory reflexes in IBD.

This review critically evaluates the emerging evidence supporting ta-VNS as an adjunctive therapy in inflammatory bowel disease, with particular emphasis on its effects on immune regulation, epithelial barrier function, microbiota-related pathways, and autonomic biomarkers. It also examines potential differences in therapeutic responses between Crohn’s disease and ulcerative colitis and identifies priorities for future clinical trials.

**Hypothesis** **1.** *ta-VNS reorganizes the gut–brain–immune axis in IBD by modulating parasympathetic activity; such modulation consequently culminates in the suppression of pro-inflammatory cytokine production, an enhancement in epithelial barrier integrity, the normalization of gut microbiota composition, and, ultimately, the amelioration of disease symptomatology*.

## 2. Methods

This article involved the execution of targeted literature searches, utilizing PubMed, PMC, and pertinent journals. The focus of these investigations encompassed “auricular vagus nerve stimulation,” “transcutaneous VNS,” “inflammatory bowel disease,” “Crohn’s,” “ulcerative colitis,” and cognate terminology. Emphasis was placed upon recent clinical trials, preclinical studies, and reviews, supplemented by key foundational studies. This review incorporated experimental colitis models, clinical trials of ta-VNS in IBD or adjacent inflammatory conditions (RA, SLE, IBS), and mechanistic papers on vagal control of inflammation. The data set was then expanded to include parameters related to stimulation, outcomes achieved, and any adverse effects. From these findings, the extrapolation of proposed mechanisms and trial designs was undertaken.

## 3. Clinical and Allied Studies

Table 1 presents a summary of the key studies of ta-VNS in IBD and related conditions. In a proof-of-concept trial of pediatric IBD, a mixture of CD and UC, with patients ranging in age from 11 to 17 years, a daily ta-VNS protocol administered for 5 min at a frequency of 5 hertz (Hz) over a period of 16 weeks resulted in clinical remission in 50% of patients with active CD and 33% of patients with active UC (. Among subjects with elevated fecal calprotectin (FC) at baseline, 64.7% achieved a ≥50% FC reduction by week 16. The median FC decreased by 81% in UC (*p* = 0.03) and 51% in CD (*p* = 0.09) [23]. No safety concerns were identified during the observation period. The present findings suggest that ta-VNS has the potential to attenuate disease activity in human IBD.

Clinical trials conducted in patient populations beyond inflammatory bowel disease additionally suggest the therapeutic efficacy of ta-VNS in the modulation of gut inflammation and the restoration of autonomic balance. In constipation-predominant IBS, a 4-week RCT (*n* = 40) indicated that the daily application of ta-VNS significantly improved global IBS symptom scores, bowel movement frequency, stool consistency, anxiety levels, depressive symptoms, and overall quality of life, as opposed to a sham intervention [21]. ta-VNS elicited an augmentation of serum acetylcholine and heart rate variability (indicative of vagal activity), concurrently mitigating nitric oxide levels [21]. Furthermore, alterations in the gut microbiota composition were observed, notably comprising increases in Bifidobacterium species and short-chain fatty acids (acetate, butyrate, and propionate) [21]. Conversely, a twelve-week open-label study (n = 27) in rheumatoid arthritis patients, which employed a wearable ta-VNS device administering 20 kHz pulses for approximately 30 min daily, demonstrated a mean DAS28-CRP reduction of –1.4 (*p* < 0.0001); notably, 37% of participants achieved clinical remission, and 57% attained a minimal clinically important improvement in HAQ-DI [20]. Similarly, a two-week randomized, double-blind, sham-controlled study in patients with functional dyspepsia (n = 36) reported improvements in dyspeptic symptoms, gastric motility, and vagal efferent activity following ta-VNS compared with sham stimulation. [22]. Collectively, these investigations suggest that ta-VNS possesses the capacity to modulate neuroimmune and microbiota factors, thereby potentially ameliorating symptomatology in inflammatory and gut–brain disorders.

These findings show that non-invasive vagal stimulation can reduce inflammatory activity and improve clinical indices in IBD models and related diseases (IBS, arthritis, SLE), thereby suggesting its potential candidacy for Inflammatory Bowel Disease treatment. A recent systematic review of VNS in gastrointestinal disorders suggested that non-invasive Vagal Nerve Stimulation may significantly improve symptoms across conditions (IBD, IBS, and other conditions) compared to sham, with low adverse events [25].

Mechanisms of action: Auricular vagus nerve stimulation is thought to modulate intestinal inflammation through interconnected neural, neuroimmune, and neuroendocrine pathways. Stimulation of the auricular branch of the vagus nerve activates afferent fibers projecting to the nucleus tractus solitarius (NTS), which functions as a major integrative center for autonomic and immune-related signals. The NTS can subsequently engage vagal efferent pathways through the dorsal motor nucleus of the vagus (DMV) and may also modulate hypothalamic–pituitary–adrenal (HPA) axis activity [1,8]. Within the context of inflammatory bowel disease (IBD), these anti-inflammatory effects can be broadly considered in terms of a local, CAIP and the systemic, canonical splenic pathway of the inflammatory reflex [12,26].

A mechanism of particular relevance to IBD is the non-canonical CAIP, which refers to spleen-independent vagal regulation of intestinal immune homeostasis [26,27]. In this pathway, vagal efferent signaling can modulate enteric neurons and thereby improve immune cells locally within the intestinal wall. Experimental evidence indicates that vagal stimulation can suppress the activation of intestinal muscularis resident macrophages through enteric neuron-mediated cholinergic signaling involving α7 nicotinic acetylcholine receptors (α7nAChRs), independently of the spleen [27]. Activation of this local pathway has been associated with reduced production of pro-inflammatory mediators, including TNF-α, and attenuation of intestinal inflammatory responses [27].

Additional evidence supports a broader vago-vagal liver–brain–gut neural arc involved in intestinal immune homeostasis [26,28]. In this circuit, hepatic vagal sensory afferents indirectly detect changes in the gut environment and transmit this information to the NTS. Subsequent vagal parasympathetic output and enteric neuronal activation stimulate muscarinic acetylcholine receptors on colonic antigen-presenting cells. This signaling increases aldehyde dehydrogenase expression and retinoic acid synthesis, thereby supporting the differentiation and maintenance of peripheral regulatory T cells in the colon [27]. Disruption of this neural arc reduces the colonic regulatory T-cell pool and increases susceptibility to experimental colitis, suggesting that direct vagal regulation of intestinal immune cells and antigen-presenting cells might be particularly relevant to IBD pathophysiology [26,27].

In parallel, VNS might engage the canonical CAIP, which represents the splenic component of the inflammatory reflex [12,29,30,31]. According to this model, vagal activity is relayed through prevertebral autonomic ganglia to the splenic nerve. Norepinephrine released from splenic nerve terminals stimulates β2-adrenergic receptors on choline acetyltransferase-positive T cells, resulting in acetylcholine release [29,30]. Acetylcholine subsequently activates α7nAChRs expressed on macrophages and suppresses the production of pro-inflammatory cytokines, particularly TNF-α [30,31]. Although this canonical pathway provides a well-established explanation for the systemic anti-inflammatory effects of VNS, its precise contribution to the control of intestinal inflammation in human IBD remains uncertain.

VNS may additionally modulate the HPA axis through afferent projections from the NTS to hypothalamic centers, potentially increasing glucocorticoid release and contributing to systemic immunomodulation [1,8]. Nevertheless, HPA axis activation should be considered a complementary mechanism rather than the principal explanation for the anti-inflammatory effects of vagal stimulation in IBD. Available experimental findings suggest that locally acting, spleen-independent vagal pathways may be more directly relevant to intestinal immune regulation than vagal effects mediated through the spleen or adrenal gland [26,27,28].

Overall, VNS is likely to exert its effects in IBD through convergent and partially overlapping mechanisms, including the non-canonical intestinal CAIP, the canonical splenic inflammatory reflex, and neuroendocrine modulation through the HPA axis. However, the relative contribution of each pathway in humans has not yet been established. Furthermore, current clinical evidence for VNS in both Crohn’s disease and ulcerative colitis is based largely on pilot and proof-of-concept studies [23,32]. Therefore, it would be premature to conclude that the CAIP or any other vagally mediated pathway is more relevant to ulcerative colitis than to Crohn’s disease.

**Figure 2:**
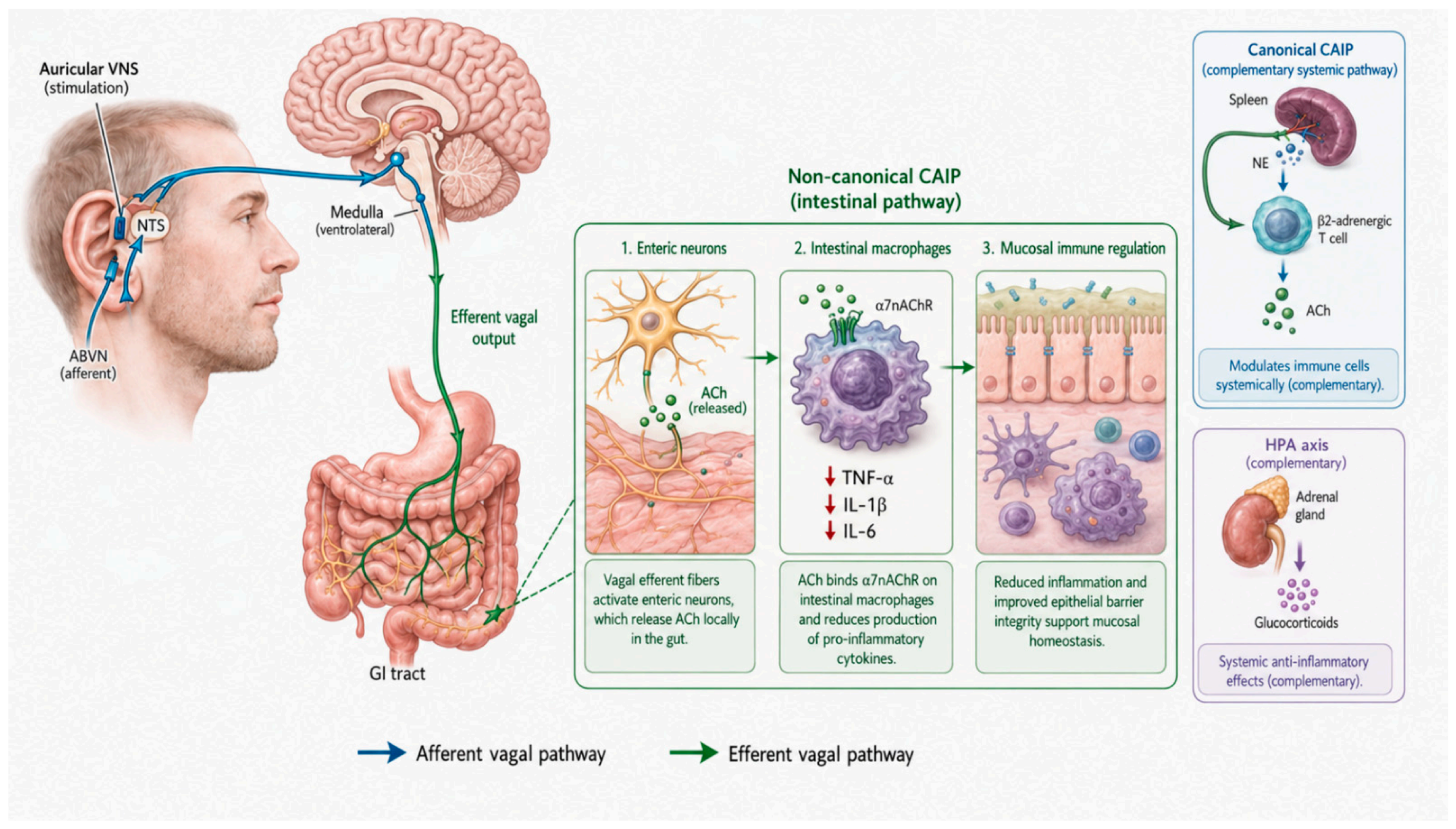
Mechanisms of Action of VNS and the Inflammatory Reflex. Red downward arrows indicate decreased production of pro-inflammatory cytokines. Abbreviations: ABVN, auricular branch of the vagus nerve; ACh, acetylcholine; CAIP, cholinergic anti-inflammatory pathway; GI, gastrointestinal; HPA, hypothalamic–pituitary–adrenal; IL-1β, interleukin-1 beta; IL-6, interleukin-6; NE, norepinephrine; NTS, nucleus tractus solitarius; TNF-α, tumor necrosis factor alpha; VNS, vagus nerve stimulation; α7nAChR, alpha-7 nicotinic acetylcholine receptor.

Transcutaneous auricular vagus nerve stimulation activates vagal afferent fibers projecting to the nucleus tractus solitarii, which coordinates downstream parasympathetic output and hypothalamic–pituitary–adrenal (HPA) axis activation. These central circuits engage multiple anti-inflammatory pathways, including the cholinergic anti-inflammatory pathway and splenic sympathetic anti-inflammatory reflexes, which may contribute to the suppression of macrophage-derived cytokines, including TNF-α, IL-1β, and IL-6. The mechanisms underlying the anti-inflammatory effects of vagus nerve stimulation are summarized in Figure 2.

Concurrently, vagal efferent signaling modulates the microbiota–gut–brain axis (MGBA), influencing gut–brain communication and indirectly affecting enteric glia and neuronal survival via microbial metabolites like short-chain fatty acids [33,34]. At the epithelial level, ta-VNS is hypothesized to reinforce intestinal barrier integrity through potential mechanisms such as the up-regulation of tight-junction proteins, decreased bacterial translocation, and reduced intestinal permeability. Restoration of sympatho-vagal balance may further contribute to the attenuation of stress-related disease flare-ups and neuropsychiatric comorbidities, potentially through effects on inflammation and the gut–brain axis. This comprehensive neuro-immune modulation underpins the therapeutic potential of VNS in managing the multifaceted pathogenesis of IBD. Further exploration into these pathways is crucial to fully elucidate the complex interplay between neural circuits, immune cells, and gut microbiota that explain the therapeutic efficacy of VNS in inflammatory bowel diseases.

**Figure 3:**
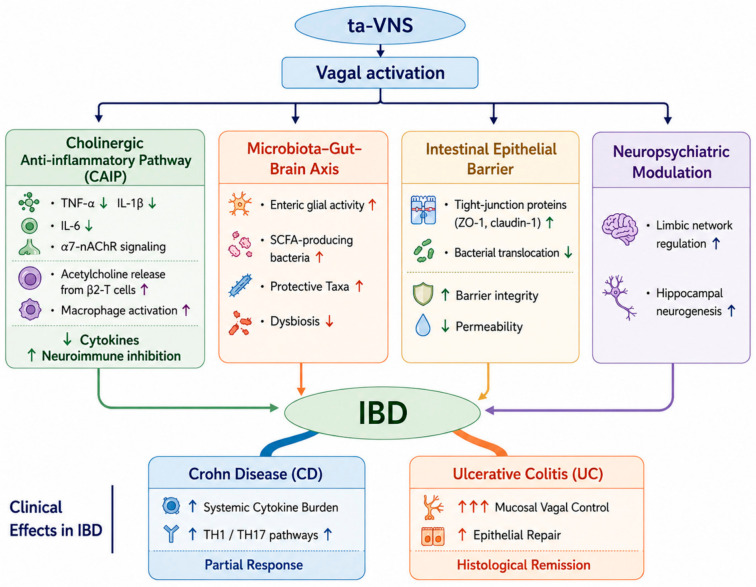
Clinical effects of ta-VNS. Note: ↑ indicates an increase or enhancement, whereas ↓ indicates a decrease or reduction. ta-VNS, transcutaneous auricular vagus nerve stimulation; CAIP, cholinergic anti-inflammatory pathway; TNF-α, tumor necrosis factor alpha; IL, interleukin; α7-nAChR, alpha-7 nicotinic acetylcholine receptor; β2-T cells, beta-2 adrenergic receptor-expressing T cells; SCFA, short-chain fatty acid; ZO-1, zonula occludens-1; IBD, inflammatory bowel disease; CD, Crohn disease; UC, ulcerative colitis; TH1, T helper 1; TH17, T helper 17.

At the clinical level, these integrated neuro-immune and metabolic effects are proposed to differentially has been shown to affect Crohn’s disease and ulcerative colitis phenotypes. In Crohn’s disease, ta-VNS may preferentially effectuate a reduction in systemic cytokine burden and Th1/Th17 signaling, potentially resulting in partial clinical responses. In ulcerative colitis, enhanced mucosal vagal control and epithelial repair are hypothesized to promote histological remission (Figure 3). This differential impact suggests that biomarker-based stratification of IBD patients could optimize therapeutic targeting and personalize aVNS interventions [35].

Barrier and microbiota: Vagal signaling exerts an influence upon both the intestinal barrier and the gut microbiome. The cholinergic anti-inflammatory pathway has been associated with the maintenance of enhanced epithelial junctional integrity. Evidence suggests that vagus nerve stimulation (VNS) may affect the reversal of stress-induced gut permeability [36]. Mogilevski and Bonaz have indicated that vagus nerve (VN) activation, occurring via enteric glia and associated neural pathways, exerts a modulatory effect upon permeability [37]. Indeed, vagal stimulation promoted an increase in the expression of tight-junction proteins within experimental IBD models [15]. Through the strengthening of the mucosal barrier, ta-VNS may impede bacterial translocation and mitigate systemic immune activation. Furthermore, the gut microbiota–brain axis appears to be mediated, at least in part, by the VN [38]. For instance, auricular stimulation elevates vagal tone, a physiological change associated with shifts toward beneficial microbiota (e.g., Bifidobacterium) and augmented levels of short-chain fatty acids (SCFAs) in IBS [21]. SCFAs possess the capacity to activate vagal afferents, potentially inducing anti-inflammatory effects. Thus, ta-VNS demonstrably offers a multi-pronged therapeutic strategy; by modulating both immunological responses and gut barrier function, it addresses critical chronic inflammatory processes observed in IBD. This multifaceted action underscores the potential of ta-VNS not merely as an anti-inflammatory agent but as a modulator of fundamental pathophysiological mechanisms underlying IBD, including gut barrier integrity and microbiota composition. It is hypothesized that ta-VNS may correct the dysbiosis observed in IBD; early data suggest that vagal modulation could enrich protective taxa and metabolites [39,40]. Moreover, this study delineated the role of neuroimmune crosstalk, which encompasses enteric glial cells and resident macrophages. Afferent vagal fibers are typically situated in close proximity to mucosal mast cells, whereas enteric neurons exhibiting cholinergic output reside adjacent to α7-positive macrophages [1]. This anatomical configuration consequently provides a foundational basis for the rapid neural regulation of gastrointestinal inflammation.

ta-VNS is proposed to (a) augment parasympathetic outflow, thereby rebalancing autonomic nervous system (ANS) homeostasis; (b) activate CAIP and hypothalamic–pituitary–adrenal (HPA) mechanisms, which may attenuate systemic TNF-α, interleukin-1 beta (IL-1β), and other pro-inflammatory cytokines; (c) ameliorate gut epithelial integrity, reducing permeability; and (d) modulate the gut microbiome–brain axis, potentially fostering the proliferation of SCFA-producing bacteria. The resultant decrease in systemic inflammation, coupled with enhanced intestinal barrier function, contributes to the overall therapeutic efficacy of ta-VNS in mitigating IBD symptomatology and progression. The collective impact of these physiological alterations may encompass a reduction in mucosal inflammation and an enhancement in tissue regeneration. A recent investigation elucidated novel mechanistic pathways: Ulloa and colleagues demonstrated that cervical VNS in murine models inhibited small ubiquitin-like modifier (SUMO)ylation, a post-translational modification, to mitigate colitis independent of interleukin-10 (IL-10)/α7 pathways; moreover, SUMO inhibitors replicated VNS effects, manifesting as reduced permeability and attenuated sepsis progression [41]. This seminal discovery elucidates additional intracellular signaling mechanisms, specifically vagally mediated inhibition of SUMOylation, which may reprogram stress-adaptive responses and restrict colitis progression independently of the canonical IL-10/α7 nicotinic cholinergic pathway. Consequently, SUMO-activating enzyme inhibitors, such as TAK-981, emerge as promising adjunctive targets for potentiating VNS efficacy across inflammatory bowel disease (IBD) models. Thus, ta-VNS may additionally leverage nascent immune programming mechanisms [26,41,42,43]. Specifically, vagal stimulation may attenuate systemic inflammation through complementary neuroimmune and neuroendocrine mechanisms, including activation of the cholinergic anti-inflammatory pathway and modulation of the hypothalamic–pituitary–adrenal axis [44]. Upon detection by vagal afferents, these pathways transmit signals to the central nervous system, which subsequently orchestrates efferent responses that may suppress pro-inflammatory cytokine release by macrophages and modulate the intestinal barrier [23,45]. This intricate neuro-immune interaction involves specific immune cell phenotypes, such as β2-adrenergic receptor-positive CD4+ T lymphocytes and α7 nicotinic acetylcholine receptor-expressing macrophages, which are deemed pivotal in the CAIP’s capacity for the inhibition of pro-inflammatory cytokines [46,47]. Through the cholinergic mediation of acetylcholine release, the vagus nerve directly inhibits both monocyte infiltration and macrophage activation, which may consequently attenuate the release of inflammatory factors such as TNF-α and IL-1β within the intestinal tract [48]. This neuromodulatory action within the intestinal milieu is mediated by enteric neurons which, subsequent to vagal activation, release acetylcholine to stimulate α7 nicotinic acetylcholine receptors on macrophages, thus inducing the inhibition of TNF-α release [49,50,51]. Beyond this direct cholinergic inhibition, VNS furthermore modulates splenic noradrenergic signaling, which subsequently modulates splenic T cells to secrete acetylcholine, thereby amplifying the anti-inflammatory cascade [52]. This intricate interplay appears to underscore the multifaceted immunomodulatory roles of vagal activation, which extend beyond direct neural pathways to encompass systemic neuro-immune circuits [53]. Furthermore, empirical investigations demonstrate that a diminution in autonomic parasympathetic tone appears to elevate IBD susceptibility and mortality, whereas vagal stimulation may ameliorate colitis via the inhibition of SUMOylation through a mechanism independent of the canonical interleukin-10 (IL-10)/α7 nicotinic cholinergic pathway [41]. This therefore underscores the inherent complexity of VNS mechanisms, suggesting the presence of both direct cholinergic anti-inflammatory effects, attributable to macrophage modulation, and broader neuro-immune axis regulation, which may involve novel intracellular pathways [54,55].

Crohn’s disease (CD) case example: The transmural, frequently small-bowel inflammation and predominant Th1/Th17 immunity characteristic of CD collectively pose distinct challenges. CD patients frequently exhibit sympathetic overactivity and vagal underactivity [56]. This observation suggests the potential benefit of augmenting vagal tone; however, the profound tissue localization and resultant fibrosis inherent in CD may potentially attenuate this effect. Notably, vagotomy in animals and humans is associated with subsequent CD onset, underscoring a protective role of the vagus nerve (VN) [57]. In the pediatric ta-VNS trial, CD patients achieved remission (fifty percent), but their median calprotectin reduction was smaller than that of UC patients [23]. In experimental models, ta-VNS had negligible efficacy in DNBS colitis [8]. Should ta-VNS primarily mitigate cytokine burden (e.g., TNF-α, IL-1β) and physiological stress, it could potentially ameliorate systemic CD symptomatology (e.g., fatigue, pain), even in the absence of complete deep ulcer resolution. This observation, therefore, indicates a prospective role for ta-VNS as an adjunctive therapeutic modality in CD, aiming to address systemic inflammation and enhance quality of life, notwithstanding the persistent difficulties associated with mucosal healing. Conversely, the anti-inflammatory effects of VNS have been widely demonstrated in various experimental models and clinical contexts, highlighting its potential to modulate the production of pro-inflammatory cytokines such as TNF, IL-1β, and IL-6. Future studies should stratify CD patients by location and phenotype (inflammatory vs. stricturing) to test this [58,59].

Ulcerative colitis (UC) case example: UC is characterized by continuous mucosal inflammation of the colon [60]. Patients with UC often have markedly low vagal tone and demonstrate a unique responsiveness to cholinergic mechanisms [60]. The efficacy of whole-body nicotine therapy and acetylcholinesterase inhibitors in attenuating colitis further supports the therapeutic potential of cholinergic modulation in UC pathogenesis. Given that α7-nicotinic acetylcholine receptors (α7-nAChRs) are critical mediators of the cholinergic anti-inflammatory pathway, their activation through vagal stimulation could significantly inhibit macrophage-mediated inflammation in the colonic mucosa, a hallmark of UC. This mechanism, involving the suppression of proinflammatory cytokine expression and stimulation of anti-inflammatory cytokine production in a macrophage-dependent manner, may position vagal nerve activation as a promising strategy for IBD treatment. Indeed, vagal activity is instrumental in influencing the mucosal immune response, promoting epithelial proliferation and controlling monocyte infiltration in the colon. In the ta-VNS pediatric trial, UC patients demonstrated significant calprotectin reductions and clinical improvements [23]. In mice, ta-VNS significantly improved DSS colitis indices, while the same treatment exhibited limited efficacy in DNBS colitis [8]. This suggests ta-VNS may be especially useful for UC by enhancing the vagal brake; ta-VNS could counteract the autonomic nervous system imbalance seen in UC. This is further supported by findings that VNS treatment in UC patients may be associated with clinical and endoscopic remission, alongside a restored vagal tone and decreased digestive pain [61]. This implies that ta-VNS may offer a targeted therapeutic approach for UC by directly addressing the underlying neuroimmunological dysregulation inherent in the disease [19]. Given the established efficacy in modulating inflammatory responses, ta-VNS may present a compelling non-pharmacological strategy for managing UC symptoms and potentially inducing sustained remission [8,62]. The mechanism underlying the differential efficacy between CD and UC may relate to distinct immunological profiles, with UC having a more pronounced cholinergic anti-inflammatory pathway dependency. Thus, optimizing ta-VNS parameters for UC patients could leverage this dependency, potentially leading to more potent anti-inflammatory outcomes compared to other IBD phenotypes [63]. The efficacy of ta-VNS in IBD may also be influenced by the presence of psychological comorbidities, given that stress can inhibit vagal anti-inflammatory effects and exacerbate IBD flare-ups [16]. Therefore, integrating psychological support alongside ta-VNS could further amplify its therapeutic potential by mitigating stress-induced vagal inhibition and improving patient outcomes. Furthermore, the interplay between the central nervous system and gut microbiota, mediated by the vagus nerve, suggests the potential for ta-VNS to exert its therapeutic effects not only through direct neural pathways but also by influencing microbial composition and function; this may reinforce mucosal integrity and reduce inflammation [64]. This intricate neuro-microbiota interaction highlights a potential avenue for personalized treatment strategies, where specific microbial signatures could predict responsiveness to ta-VNS. This perspective underscores the necessity of biomarker-driven approaches to tailor ta-VNS interventions, moving beyond a one-size-fits-all model toward stratified therapies that consider individual patient endotypes [64,65,66]. Such approaches would necessitate the identification of specific molecular and physiological indicators that predict therapeutic success or failure, allowing for the optimization of stimulation parameters and treatment duration.

## 4. Limitations and Knowledge Gaps

Current evidence for ta-VNS in IBD is limited by limited study cohorts and short durations. For instance, a pediatric IBD trial encompassed twenty-two subjects (ten with Crohn’s Disease and twelve with Ulcerative Colitis) presenting with active disease [23]. Presently, the establishment of large-scale, protracted ta-VNS trials within the adult IBD population remains unobserved. The durability of therapeutic effects remains equivocal, as does the potential for diminishing efficacy of stimulation across extended periods. Moreover, the therapeutic outcomes of ta-VNS may exhibit variability contingent upon individual anatomical distinctions (e.g., vagal innervation patterns of the ear) or specific disease phenotypes. Furthermore, the exact neural circuits in human IBD remain incompletely delineated; for example, the balance between direct enteric neuron and splenic pathways remains debated [65]. The potential for substantial placebo effects within neuromodulation trials, thus, necessitates the implementation of rigorous control methodologies. Finally, while emphasis may be placed upon parasympathetic mechanisms, other neural pathways (e.g., splanchnic sympathetic reflexes) may concurrently exert contributory effects, thereby warranting further investigation.

In summary, converging data suggest that ta-VNS can attenuate gut inflammation through the mediation of multifaceted neuroimmune pathways. By engaging the cholinergic anti-inflammatory reflex, potentiating the HPA axis, and improving barrier and microbiota health, ta-VNS could potentially complement extant IBD therapeutics, evidencing negligible associated morbidity. Notably, Ulcerative Colitis (UC) may derive a greater therapeutic benefit than Crohn’s Disease (CD), considering the documented vagal deficits and nicotine effects observed within UC [61,65]. The implementation of clinical trials for ta-VNS in IBD is thus advocated; feasible endpoints for such investigations encompass symptomatic remission rates, endoscopic scores, and objective biomarkers (e.g., calprotectin, heart rate variability). Should its efficacy be substantiated, ta-VNS would introduce a novel non-pharmacological and non-surgical modality into the IBD armamentarium.

## 5. Conclusions

Transcutaneous auricular vagus nerve stimulation represents a promising non-invasive adjunctive strategy for inflammatory bowel disease through its potential effects on neuroimmune regulation and intestinal homeostasis [67,68,69]. However, the current clinical evidence remains limited and preliminary, and no firm conclusions can yet be drawn regarding efficacy or disease-specific responses. Future well-designed randomized controlled trials should establish standardized stimulation parameters, identify predictive biomarkers, and determine the patient subgroups most likely to benefit from this strategy.

## Figures and Tables

**Table 1:** Effects of ta-VNS on inflammatory, neural, and gut-barrier-related outcomes in experimental and clinical models of intestinal inflammation.

Study	Human Disease/Animal Model	ta-VNS Protocol	Key Outcomes	Sample Size
Sahn et al. [23]	Human pediatric IBD (CD and UC)	5 min/day, 5 Hz, 16 weeks	50% of active CD and 33% of active UC patients achieved remission; 64.7% had ≥50% fecal calprotectin reduction	22 participants
Hesampour et al. [8]	Animal murine colitis (Dextran Sodium Sulfate [DSS]-induced, modeling Ulcerative Colitis, and Dinitrobenzenesulfonic Acid [DNBS]-induced, modeling Crohn’s Disease)	10 min/day for 6 days (DSS model); 10 min/day for 5 days (DNBS model), 20 Hz	In DSS model: ↓DAI and histological scores, ↓IL-1β, ↓TNF-α, ↑TGF-β, ↑IL-10 (anti-inflammatory); no effect in DNBS model	30 mice
Soylu et al. [24]	Animal rattus norvegicus 2,4,6-Trinitrobenzenesulfonic Acid (TNBS)-induced Colitis (an Inflammatory Bowel Disease model)	30 min × 2/day, duration 10 days	↑enteric synaptic proteins (neuroligin-1, neurexin-2α) and improved colonic inflammation compared to no stimulation	36 rats
Liu et al. [21]	Human constipation-predominant IBS	30 min/day, 4 weeks	Significant improvements in IBS severity, bowel habits, anxiety/depression; ↑serum ACh (*p* = 0.005), ↑vagal tone, ↑Bifidobacteria and SCFAs	42 participants
Marsal et al. [20]	Human active Rheumatoid Arthritis in human participants	~30 min/day (20 kHz pulses), 12 weeks	Mean DAS28-CRP ↓1.4 (*p* < 0.0001); 37% reached remission; HAQ-DI Δ–0.5 (*p* < 0.0001)	30 participants
Zhu et al. [22]	Human functional dyspepsia in human subjects	ta-VNS versus sham, for 2 weeks	ta-VNS vs. sham: improved dyspeptic symptoms, gastric accommodation, gastric slow waves, and vagal efferent activity; reduced depression and anxiety scores	36 participants

Note: ↑ indicates an increase, whereas ↓ indicates a decrease.

## Data Availability

No new data were created or analyzed in this study. Data sharing is not applicable to this article.

## References

[1] Fornaro R., Actis G.C., Caviglia G.P., Pitoni D., Ribaldone D.G. (2022). Inflammatory Bowel Disease: Role of Vagus Nerve Stimulation. J. Clin. Med..

[2] Bonaz B. (2023). Non-Invasive Vagus Nerve Stimulation: The Future of Inflammatory Bowel Disease Treatment?. Bioelectron. Med..

[3] Cao H., Diao J., Liu H., Liu S., Liu J., Yuan J., Lin J. (2022). The Pathogenicity and Synergistic Action of Th1 and Th17 Cells in Inflammatory Bowel Diseases. Inflamm. Bowel Dis..

[4] Lee S., Kwon J.E., Cho M. (2018). Immunological Pathogenesis of Inflammatory Bowel Disease. Intest. Res..

[5] Zhang Y.Z., Li Y.Y. (2014). Inflammatory Bowel Disease: Pathogenesis. World J. Gastroenterol..

[6] Caioni G., Viscido A., d’Angelo M., Panella G., Castelli V., Merola C., Frieri G., Latella G., Cimini A., Benedetti E. (2021). Inflammatory Bowel Disease: New Insights into the Interplay between Environmental Factors and PPARγ. Int. J. Mol. Sci..

[7] Hafez M.M., Bahçecioğlu İ.H., Yalnız M., Kouta K.A., Tawheed A. (2025). Future of Inflammatory Bowel Disease Treatment: A Review of Novel Treatments beyond Guidelines. World J. Methodol..

[8] Hesampour F., Tshikudi D.M., Bernstein Ç.N., Ghia J. (2024). Exploring the Efficacy of Transcutaneous Auricular Vagus Nerve Stimulation (taVNS) in Modulating Local and Systemic Inflammation in Experimental Models of Colitis. Bioelectron. Med..

[9] van der Valk M.E., Mangen M.J., Severs M., van der Have M., Dijkstra G., van Bodegraven A.A., Fidder H.H., de Jong D.J., Pierik M., van der Woude C.J. (2015). Comparison of Costs and Quality of Life in Ulcerative Colitis Patients with an Ileal Pouch-Anal Anastomosis, Ileostomy and Anti-TNFα Therapy. J. Crohns Colitis.

[10] Burisch J., Vardi H., Schwartz D., Friger M., Kiudelis G., Kupčinskas J., Fumery M., Gower-Rousseau C., Lakatos L., Lakatos P.L. (2020). Health-Care Costs of Inflammatory Bowel Disease in a Pan-European, Community-Based, Inception Cohort during 5 Years of Follow-up: A Population-Based Study. Lancet Gastroenterol. Hepatol..

[11] Na S., Kim Y.S. (2022). Management of Inflammatory Bowel Disease beyond Tumor Necrosis Factor Inhibitors: Novel Biologics and Small-Molecule Drugs. Korean J. Intern. Med..

[12] Tracey K.J. (2002). The Inflammatory Reflex. Nature.

[13] Pavlov V.A., Tracey K.J. (2012). The Vagus Nerve and the Inflammatory Reflex—Linking Immunity and Metabolism. Nat. Rev. Endocrinol..

[14] Huston J.M. (2012). The Vagus Nerve and the Inflammatory Reflex: Wandering on a New Treatment Paradigm for Systemic Inflammation and Sepsis. Surg. Infect..

[15] Bonaz B., Sinniger V., Pellissier S. (2017). Vagus Nerve Stimulation: A New Promising Therapeutic Tool in Inflammatory Bowel Disease. J. Intern. Med..

[16] Ge L., Liu S., Li S., Yang J., Hu G., Xu C., Song W. (2022). Psychological Stress in Inflammatory Bowel Disease: Psychoneuroimmunological Insights into Bidirectional Gut–Brain Communications. Front. Immunol..

[17] Pellissier S., Dantzer C., Canini F., Mathieu N., Bonaz B. (2009). Psychological Adjustment and Autonomic Disturbances in Inflammatory Bowel Diseases and Irritable Bowel Syndrome. Psychoneuroendocrinology.

[18] Lindgren S., Lilja B., Rosén I., Sundkvist G. (1991). Disturbed Autonomic Nerve Function in Patients with Crohn’s Disease. Scand. J. Gastroenterol..

[19] Pikov V. (2023). Vagus Nerve Stimulation and Sacral Nerve Stimulation for Inflammatory Bowel Disease: A Systematic Review. J. Transl. Gastroenterol..

[20] Marsal S., Corominas H., de Agustín J.J., Pérez-García C., López-Lasanta M., Borrell H., Reina D., Sanmartí R., Narváez J., Franco-Jarava C. (2021). Non-Invasive Vagus Nerve Stimulation for Rheumatoid Arthritis: A Proof-of-Concept Study. Lancet Rheumatol..

[21] Liu J., Lv C., Yin M., Zhu M., Wang B., Tian J., Hashimoto K., Yu Y. (2024). Efficacy and Safety of Transcutaneous Auricular Vagus Nerve Stimulation in Patients with Constipation-Predominant Irritable Bowel Syndrome: A Single-Center, Single-Blind, Randomized Controlled Trial. Am. J. Gastroenterol..

[22] Zhu Y., Xu F., Lu D., Rong P., Cheng J., Li M., Gong Y., Sun C., Wei W., Lin L. (2021). Transcutaneous auricular vagal nerve stimulation improves functional dyspepsia by enhancing vagal efferent activity. Am. J. Physiol. Gastrointest. Liver Physiol..

[23] Sahn B., Pascuma K., Kohn N., Tracey K.J., Markowitz J. (2023). Transcutaneous Auricular Vagus Nerve Stimulation Attenuates Inflammatory Bowel Disease in Children: A Proof-of-Concept Clinical Trial. Bioelectron. Med..

[24] Soylu A., Alim E., Akarca Dizakar S.O., Atalar K., Bahcelioglu M. (2025). Is Transauricular Vagus Nerve Stimulation the Spark for Modulation of Synaptic Alterations in Ulcerative Colitis?. Bratisl. Med. J..

[25] Veldman F., Hawinkels K., Keszthelyi D. (2025). Efficacy of Vagus Nerve Stimulation in Gastrointestinal Disorders: A Systematic Review. Gastroenterol. Rep..

[26] de Araujo A., de Lartigue G. (2020). Non-canonical cholinergic anti-inflammatory pathway in IBD. Nat. Rev. Gastroenterol. Hepatol..

[27] Matteoli G., Gomez-Pinilla P.J., Nemethova A., Di Giovangiulio M., Cailotto C., van Bree S.H., Michel K., Tracey K.J., Schemann M., Boesmans W. (2014). A distinct vagal anti-inflammatory pathway modulates intestinal muscularis resident macrophages independent of the spleen. Gut.

[28] Teratani T., Mikami Y., Nakamoto N., Suzuki T., Harada Y., Okabayashi K., Hagihara Y., Taniki N., Kohno K., Shibata S. (2020). The liver-brain-gut neural arc maintains the T_reg_ cell niche in the gut. Nature.

[29] Rosas-Ballina M., Ochani M., Parrish W.R., Ochani K., Harris Y.T., Huston J.M., Chavan S., Tracey K.J. (2008). Splenic nerve is required for cholinergic antiinflammatory pathway control of TNF in endotoxemia. Proc. Natl. Acad. Sci. USA.

[30] Rosas-Ballina M., Olofsson P.S., Ochani M., Valdés-Ferrer S.I., Levine Y.A., Reardon C., Tusche M.W., Pavlov V.A., Andersson U., Chavan S. (2011). Acetylcholine-synthesizing T cells relay neural signals in a vagus nerve circuit. Science.

[31] Borovikova L.V., Ivanova S., Zhang M., Yang H., Botchkina G.I., Watkins L.R., Wang H., Abumrad N., Eaton J.W., Tracey K.J. (2000). Vagus nerve stimulation attenuates the systemic inflammatory response to endotoxin. Nature.

[32] Bonaz B., Sinniger V., Hoffmann D., Clarençon D., Mathieu N., Dantzer C., Vercueil L., Picq C., Trocmé C., Faure P. (2016). Chronic vagus nerve stimulation in Crohn’s disease: A 6-month follow-up pilot study. Neurogastroenterol. Motil..

[33] Vicentini F.A., Keenan C.M., Wallace L.E., Woods C., Cavin J.B., Flockton A.R., Macklin W.B., Belkind-Gerson J., Hirota S.A., Sharkey K.A. (2021). Intestinal Microbiota Shapes Gut Physiology and Regulates Enteric Neurons and Glia. Microbiome.

[34] Săvulescu-Fiedler I., Benea Ș., Căruntu C., Nancoff A.-S., Homentcovschi C., Bucurica S. (2025). Rewiring the Brain Through the Gut: Insights into Microbiota–Nervous System Interactions. Curr. Issues Mol. Biol..

[35] Cirillo G., Negrete-Diaz F., Yucumá D., Virtuoso A., Korai S.A., Luca C.D., Kaniušas E., Papa M., Panetsos F. (2022). Vagus Nerve Stimulation: A Personalized Therapeutic Approach for Crohn’s and Other Inflammatory Bowel Diseases. Cells.

[36] Breit S., Kupferberg A., Rogler G., Hasler G. (2018). Vagus Nerve as Modulator of the Brain–Gut Axis in Psychiatric and Inflammatory Disorders. Front. Psychiatry.

[37] Mogilevski T., Rosella S., Aziz Q., Gibson P.R. (2022). Transcutaneous Vagal Nerve Stimulation Protects against Stress-induced Intestinal Barrier Dysfunction in Healthy Adults. Neurogastroenterol. Motil..

[38] Bonaz B., Bazin T., Pellissier S. (2018). The Vagus Nerve at the Interface of the Microbiota-Gut-Brain Axis. Front. Neurosci..

[39] Kolcun J.P.G., Burks S.S., Wang M.Y. (2017). Vagal Nerve Stimulation for Inflammatory Bowel Disease. Neurosurgery.

[40] Goggins E., Mitani S., Tanaka S. (2022). Clinical Perspectives on Vagus Nerve Stimulation: Present and Future. Clin. Sci..

[41] Youssef A., Rehman A.U., Elebasy M., Roper J., Sheikh S.Z., Karhausen J., Yang W., Ulloa L. (2024). Vagal Stimulation Ameliorates Murine Colitis by Regulating SUMOylation. Sci. Transl. Med..

[42] Karhausen J., Ulloa L., Yang W. (2021). SUMOylation Connects Cell Stress Responses and Inflammatory Control: Lessons from the Gut as a Model Organ. Front. Immunol..

[43] Ma X., Li M., Qi G., Wei L., Zhang D. (2024). SUMOylation at the Crossroads of Gut Health: Insights into Physiology and Pathology. Cell Commun. Signal..

[44] Pavlov V.A., Tracey K.J. (2017). Neural regulation of immunity: Molecular mechanisms and clinical translation. Nat. Neurosci..

[45] Bonaz B. (2022). Anti-inflammatory Effects of Vagal Nerve Stimulation with a Special Attention to Intestinal Barrier Dysfunction. Neurogastroenterol. Motil..

[46] You X., Zhang H., Han X., Wang F., Zhuang P., Zhang Y. (2021). Intestinal Mucosal Barrier Is Regulated by Intestinal Tract Neuro-Immune Interplay. Front. Pharmacol..

[47] Wang R.C., Alam M.J., Lan X., Li F., Chen J.D. (2025). Efficacy and Mechanisms of Neuromodulation in the Treatment of Irritable Bowel Syndrome. Bioelectron. Med..

[48] Zhu Y., Duan S., Wang M., Deng Z., Li J. (2022). Neuroimmune Interaction: A Widespread Mutual Regulation and the Weapons for Barrier Organs. Front. Cell Dev. Biol..

[49] Alam M.J., Zhao T., Wiley J.W., Chen J.D.Z. (2024). Comparisons of Different Electrical Stimulation Modalities for Treating Visceral Pain in a Rodent Model of Irritable Bowel Syndrome. Bioelectron. Med..

[50] Tan C., Yan Q., Ma Y., Fang J., Yang Y. (2022). Recognizing the Role of the Vagus Nerve in Depression from Microbiota-Gut Brain Axis. Front. Neurol..

[51] Shi X., Hu Y., Zhang B., Li W., Chen J.D., Liu F. (2021). Ameliorating Effects and Mechanisms of Transcutaneous Auricular Vagal Nerve Stimulation on Abdominal Pain and Constipation. JCI Insight.

[52] Wang S., Zhou S., Han Z., Yu B., Xu Y., Lin Y., Chen Y., Zi J., Li Y., Cao Q. (2024). From Gut to Brain: Understanding the Role of Microbiota in Inflammatory Bowel Disease. Front. Immunol..

[53] Brinkman D.J., Simon T., Hove A.S.T., Zafeiropoulou K., Welting O., van Hamersveld P.H.P., Willemze R., Yim A.Y.F.L., Verseijden C., Hakvoort T.B.M. (2022). Electrical Stimulation of the Splenic Nerve Bundle Ameliorates Dextran Sulfate Sodium-Induced Colitis in Mice. J. Neuroinflammation.

[54] Zheng L., Duan S.-L. (2025). Neuroimmune Interactions in Inflammatory Bowel Disease: Role of Intestinal Macrophages and the Cholinergic Pathway. World J. Gastroenterol..

[55] Mikami Y., Tsunoda J., Kiyohara H., Taniki N., Teratani T., Kanai∥ T. (2021). Vagus Nerve-Mediated Intestinal Immune Regulation: Therapeutic Implications of Inflammatory Bowel Diseases. Int. Immunol..

[56] Zawadka-Kunikowska M., Słomko J., Kłopocka M., Liebert A., Tafil-Klawe M., Klawe J.J., Newton J.L., Zalewski P. (2018). Cardiac and Autonomic Function in Patients with Crohn’s Disease during Remission. Adv. Med. Sci..

[57] Liu B., Wanders A., Wirdefeldt K., Sjölander A., Sachs M.C., Eberhardson M., Ye W., Ekbom A., Olén O., Ludvigsson J.F. (2020). Vagotomy and Subsequent Risk of Inflammatory Bowel Disease: A Nationwide Register-based Matched Cohort Study. Aliment. Pharmacol. Ther..

[58] Hesampour F., Bernstein Ç.N., Ghia J. (2023). Brain-Gut Axis: Invasive and Noninvasive Vagus Nerve Stimulation, Limitations, and Potential Therapeutic Approaches. Inflamm. Bowel Dis..

[59] Catalán-Serra I., Ricanek P., Grimstad T. (2023). “Out of the Box” New Therapeutic Strategies for Crohn’s Disease: Moving beyond Biologics. Rev. Esp. Enfermedades Dig..

[60] Pellissier S., Dantzer C., Mondillon L., Trocmé C., Gauchez A., Ducros V., Mathieu N., Toussaint B., Fournier A., Canini F. (2014). Relationship between Vagal Tone, Cortisol, TNF-Alpha, Epinephrine and Negative Affects in Crohn’s Disease and Irritable Bowel Syndrome. PLoS ONE.

[61] Serafini M.A., Paz A.H.d.R., Schneider N. (2021). Cholinergic Immunomodulation in Inflammatory Bowel Diseases. Brain Behav. Immun. Health.

[62] Bahadir S., Robinson J.T., Morse L.R., Yoo P.B., Kern R., Makowski N.S., Kozai T.D.Y., Fudim M., Barbe M.F., Lim H.H. (2026). The Sixth Bioelectronic Medicine Summit: Neurotechnologies for Individuals and Communities. Bioelectron. Med..

[63] Sahn B., Pascuma K., Tracey K., Markowitz J. (2021). P072 Non-invasive Vagal Nerve Stimulation to Treat Crohn Disease and Ulcerative Colitis in Children and Young Adults: A Proof-of-Concept Clinical Trial. Am. J. Gastroenterol..

[64] Meroni E., Stakenborg N., Gomez-Pinilla P.J., Stakenborg M., Aguilera-Lizarraga J., Florens M., Delfini M., de Simone V., De Hertogh G., Goverse G. (2021). Vagus Nerve Stimulation Promotes Epithelial Proliferation and Controls Colon Monocyte Infiltration During DSS-Induced Colitis. Front. Med..

[65] Duan H., Cai X., Luan Y., Yang S., Yang J., Dong H., Zeng H., Shao L. (2021). Regulation of the Autonomic Nervous System on Intestine. Front. Physiol..

[66] Yang D., Almanzar N., Chiu I.M. (2023). The Role of Cellular and Molecular Neuroimmune Crosstalk in Gut Immunity. Cell. Mol. Immunol..

[67] Rangari G., Keshetty N.S., Meka P., Nalam R., Bhupalwar S.K., Sawaiwar R.Y., Khan A.Z., Palakodeti S. (2025). S1626 Evaluating the Role of Neuromodulatory Techniques on Clinical Outcomes in Inflammatory Bowel Disease: A Systematic Review. Am. J. Gastroenterol..

[68] Dilixiati S., Yan J., Qingzhuoga D., Song G., Tu L. (2024). Exploring Electrical Neuromodulation as an Alternative Therapeutic Approach in Inflammatory Bowel Diseases. Medicina.

[69] Thompson S.L., O’Leary G.H., Austelle C.W., Gruber E., Kahn A.T., Manett A.J., Short B., Badran B.W. (2021). A Review of Parameter Settings for Invasive and Non-invasive Vagus Nerve Stimulation (VNS) Applied in Neurological and Psychiatric Disorders. Front. Neurosci..

